# From Mystery to Clarity: Uncovering the Possible Cause of Hepatitis Outbreak in Children

**DOI:** 10.7759/cureus.38388

**Published:** 2023-05-01

**Authors:** Ashritha Rao, Mayur B Wanjari, Roshan Prasad, Pratiksha K Munjewar, Ranjana Sharma

**Affiliations:** 1 Pediatrics, Jawaharlal Nehru Medical College, Datta Meghe Institute of Higher Education and Research, Wardha, IND; 2 Research and Development, Jawaharlal Nehru Medical College, Datta Meghe Institute of Higher Education and Research, Wardha, IND; 3 Medicine and Surgery, Jawaharlal Nehru Medical College, Datta Meghe Institute of Higher Education and Research, Wardha, IND; 4 Medical Surgical Nursing, Smt. Radhikabai Meghe Memorial College of Nursing, Datta Meghe Institute of Higher Education and Research, Wardha, IND

**Keywords:** risk factors, prevention, children, outbreaks, hepatitis a

## Abstract

Hepatitis A is a viral infection that can cause liver inflammation and damage. Hepatitis A outbreaks in children are of particular concern due to the potential long-term health effects they can have. In recent years, several outbreaks of hepatitis A have been reported worldwide, affecting mainly children and young adults. Since 2016, hepatitis A outbreaks have been reported in 37 states of the United States alone, involving approximately 44,650 cases, 27,250 hospitalizations, and 415 deaths as of September 23, 2022. The epidemiology of hepatitis A outbreaks in children is complex and multifactorial, with various risk factors such as poor sanitation and hygiene practices, crowded living conditions, low socioeconomic status, lack of vaccination, and travel to endemic areas. Investigations of outbreaks involve identifying suspected cases, laboratory testing, contact tracing, and investigation of possible sources of infection.

Contaminated food and water, poor sanitation and hygiene procedures, intimate contact with infected people, and environmental variables are all potential causes of outbreaks in children. Preventive measures include vaccination, improving sanitation and hygiene practices, food safety and inspection, and health education and community outreach programs. Understanding the epidemiology of hepatitis A outbreaks in children and the risk factors associated with infection is essential for developing effective preventive strategies and reducing the global burden of this disease.

## Introduction and background

Hepatitis is a viral infection that affects millions of people worldwide, causing liver inflammation and, in some cases, liver damage. This disease can be caused by different viruses, including hepatitis A, B, C, D, and E [1]. Among these, hepatitis A is particularly concerning as it can cause acute liver disease, which can be severe and can lead to long-term health complications, especially in children [2,3].

Hepatitis outbreaks in children are of great concern due to the potential long-term effects on their health. Children infected with the hepatitis virus may experience symptoms, such as fever, fatigue, abdominal pain, and jaundice, which can be debilitating and significantly impact their growth and development [2]. In addition, hepatitis outbreaks can cause social and economic disruption, especially in low-income countries where access to healthcare facilities and resources is limited [2,3].

The Centers for Disease Control and Prevention (CDC) reports that hepatitis A is usually transmitted through contaminated food or water or close contact with an infected person. In 2018, an outbreak of hepatitis A was reported in several states in the United States, affecting mostly children and young adults. The outbreak was traced back to consuming contaminated frozen strawberries from a particular brand. The investigation into the hepatitis A outbreak highlights the importance of identifying the possible sources of infection and taking prompt action to prevent further spread [4,5].

In this paper, we aim to uncover the possible cause of a hepatitis outbreak in children in a primary school in a rural area of a developing country, where access to clean water and sanitation facilities is limited. The outbreak affected several children who presented with symptoms such as fever, fatigue, abdominal pain, and jaundice. Our objective is to move from mystery to clarity by investigating the possible sources of infection, identifying the contributing factors, and proposing preventive measures to reduce the risk of future outbreaks.

Investigating hepatitis outbreaks is crucial for preventing the spread of this disease and protecting public health. Understanding the transmission dynamics of the virus and the risk factors associated with infection can help develop effective preventive strategies. In this paper, we will explore the possible causes of the hepatitis outbreak in children and discuss the measures that can be taken to prevent future outbreaks. We hope that the findings of our study will contribute to understanding the epidemiology of hepatitis A and inform public health policies and interventions aimed at controlling the spread of this disease.

## Review

Methodology

**Figure 1:**
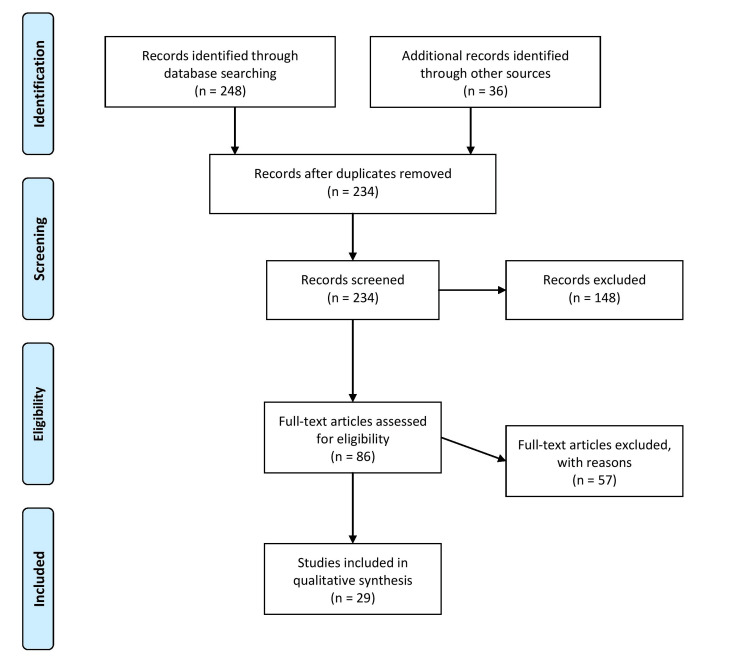
The selection process of articles used in this study. Adopted from the Preferred Reporting Items for Systematic Reviews and Meta-Analyses (PRISMA).

The methodology for the review article involves a comprehensive literature search of published studies, reviews, and reports on the epidemiology, investigations, possible sources, and preventive measures for hepatitis A outbreaks in children. The databases used for the literature search included PubMed, Scopus, Web of Science, and Google Scholar, using a combination of relevant keywords. Inclusion criteria for the selection of studies were limited to those that focused on hepatitis A outbreaks in children and were published in English between 2000 and 2022. Exclusion criteria included studies that were irrelevant to the topic, duplicates, and those not available in full text. The selected studies were critically appraised, and data were synthesized and presented narratively. Figure 1 describes the selection process of articles used in our study.

Epidemiology of hepatitis A outbreaks in children

Global Burden of Hepatitis A

Hepatitis A is a highly contagious viral infection that affects millions of people worldwide, causing inflammation and damage to the liver. According to the World Health Organization (WHO), there are estimated to be approximately 1.5 million cases of hepatitis A annually, with a wide variation in incidence rates across different regions of the world. Most cases are reported in low- and middle-income countries, particularly in areas with poor sanitation and hygiene infrastructure [6].

Endemic transmission of hepatitis A is common in these regions, meaning the virus is constant throughout the year. In contrast, high-income countries generally experience sporadic cases or outbreaks of the disease. This is largely due to the availability of clean water, adequate sanitation, and vaccination programs that have helped to reduce the burden of hepatitis A in these countries [7].

The burden of hepatitis A is particularly high among children, who are at a greater risk of infection and severe disease. In some regions, up to 90% of the population may have been infected with the virus by age 10 [8,9]. In addition to the direct health impact of the disease, hepatitis A outbreaks can have significant economic and social consequences, particularly in low-income settings where access to healthcare and resources may be limited. Overall, the global burden of hepatitis A highlights the need for continued efforts to improve sanitation and hygiene infrastructure, increase access to vaccination, and enhance surveillance and outbreak response measures [9,10].

Epidemiology of Hepatitis A Outbreaks in Children

Hepatitis A outbreaks in children represent a significant public health concern due to the potential long-term effects on their health [11]. Children infected with the hepatitis A virus can experience acute liver disease, which can be severe and lead to long-term health complications, such as chronic liver disease or liver cancer. In recent years, several hepatitis A outbreaks have been reported in various parts of the world, affecting mostly children and young adults [1,8].

The epidemiology of hepatitis A outbreaks in children is complex and multifactorial, involving various risk factors such as population density, sanitation facilities, and hygiene practices. Outbreaks can occur in different settings, including schools, childcare centers, and households. Children in crowded and low-resource settings, where inadequate sanitation and hygiene facilities are particularly vulnerable to hepatitis A infection [5,6,10]. Furthermore, close contact with infected individuals and consuming contaminated food or water are common transmission routes for hepatitis A outbreaks in children. However, outbreaks have also been reported in high-income countries, particularly in settings with a high concentration of susceptible individuals, such as schools and daycare centers [12]. It is worth noting that although the incidence of hepatitis A outbreaks has declined globally in recent years, periodic outbreaks continue to occur, particularly in vulnerable populations, such as children.

Risk Factors for Hepatitis A Outbreaks in Children

Several risk factors have been identified for hepatitis A outbreaks in children. These include the following.

Poor sanitation and hygiene practices: Hepatitis A is commonly transmitted through the fecal-oral route, and poor sanitation and hygiene practices can contribute to the spread of the virus. Inadequate water supply and sanitation facilities, lack of handwashing, and poor food hygiene are among the major risk factors for hepatitis A outbreaks in children [6].

Crowded living conditions: Overcrowding and close contact with infected individuals can increase the risk of transmission of hepatitis A, particularly in settings such as schools and childcare centers [4,5].

Low socioeconomic status: Children from low-income families may be at a higher risk of hepatitis A infection due to inadequate access to clean water and sanitation facilities and limited healthcare resources [9].

Lack of vaccination: Vaccination is an effective preventive measure for hepatitis A, and children who have not been vaccinated are at a higher risk of infection. In some countries, routine hepatitis A vaccination is not included in the national immunization schedule, which can contribute to the occurrence of outbreaks [7].

Travel to endemic areas: Children who travel to endemic areas may be at a higher risk of hepatitis A infection, particularly if they consume contaminated food or water or have close contact with infected individuals. Understanding the epidemiology of hepatitis, A outbreaks in children and the risk factors associated with infection is crucial for developing effective preventive strategies [4-6].

Investigations of hepatitis A outbreaks in children

Identification of Suspected Cases

The first step in investigating a hepatitis A outbreak in children is to identify suspected cases. In some cases, outbreaks may be identified through routine surveillance systems, while in others, clusters of cases may be reported by healthcare providers or parents. It is important to have a high level of suspicion for hepatitis A in any child presenting with acute liver disease symptoms, including jaundice, fever, and abdominal pain [4,6].

Laboratory Testing and Confirmation of Hepatitis A Cases

Laboratory testing is crucial for confirming cases of hepatitis A and determining the scope of an outbreak. The most used test for hepatitis A is the detection of immunoglobulin M (IgM) antibodies to the virus in blood samples. In outbreak situations, all suspected cases should be tested for hepatitis A, and confirmed cases should be reported to public health authorities [2,7].

Contact Tracing and Investigation of Possible Sources of Infection

Contact tracing is an important component of outbreak investigations, as it helps to identify other individuals who may have been exposed to the virus and may be at risk of infection. In the case of hepatitis, A outbreaks in children, contact tracing may involve identifying other children in the same school or childcare center, family members, and close contacts of infected individuals.

Investigation of possible sources of infection is also important in identifying the cause of an outbreak and preventing further spread. Possible sources of infection may include contaminated food or water or close contact with infected individuals. Public health authorities may conduct environmental sampling of food or water sources and inspect facilities such as schools and childcare centers to identify potential sources of contamination [2,10,11].

Analysis of Data and Identification of Risk Factors Associated With Infection

Analysis of outbreak data can provide valuable insights into the risk factors associated with hepatitis A infection in children. Data analysis may include the identification of demographic and clinical characteristics of cases, as well as the identification of common exposures or behaviors associated with infection. This information can help to inform public health interventions and prevention strategies [4-6].

Possible sources of hepatitis A outbreaks in children

Various factors, including contaminated food and water sources, poor sanitation and hygiene practices, close contact with infected individuals, and environmental factors, can cause hepatitis A outbreaks in children.

Contaminated Food and Water Sources

Contaminated food and water sources commonly cause hepatitis A outbreaks, particularly in areas with poor sanitation and hygiene practices. Hepatitis A virus can survive in water and on surfaces for extended periods and can be transmitted by consuming contaminated food or water. In some cases, outbreaks have been linked to consuming raw or undercooked shellfish, fruits, and vegetables contaminated with fecal matter or drinking water [13,14].

Poor Sanitation and Hygiene Practices

Poor sanitation and hygiene practices, such as inadequate hand washing, can also contribute to the spreading of hepatitis A in children. Hepatitis A virus is highly contagious and can be easily transmitted from person to person through close contact or the fecal-oral route. In areas with poor sanitation and hygiene practices, such as limited access to clean water or inadequate sewage systems, the risk of hepatitis A transmission can be significantly higher [15,16].

Close Contact With Infected Individuals

Close contact with infected individuals, particularly in crowded settings such as schools or childcare centers, can also increase the risk of hepatitis A transmission in children. Hepatitis A virus is most contagious two weeks before and one week after the onset of symptoms but can also be spread by asymptomatic individuals. Children not vaccinated against hepatitis A may be at higher risk of infection if they come into close contact with infected individuals [17,18].

Environmental Factors

Environmental factors, such as floods or natural disasters, can also contribute to the spread of hepatitis A in children. In areas affected by natural disasters, access to clean water and sanitation facilities may be limited, increasing the risk of virus transmission. Similarly, overcrowding in shelters or other emergency settings can increase the risk of hepatitis A transmission [19].

Preventive measures for hepatitis A outbreaks in children

Preventing hepatitis, A outbreaks in children requires a multifaceted approach that includes vaccination, improving sanitation and hygiene practices, food safety and inspection, and health education and community outreach programs [20-22].

Vaccination

Vaccination is the most effective method of preventing hepatitis A in children. The hepatitis A vaccine is safe, highly effective, and recommended for children in many countries. The vaccine can be administered as part of routine childhood immunizations or as a catch-up vaccine for older children who have not been previously vaccinated. Vaccination not only protects children from the immediate health risks of hepatitis A but also provides long-term protection against future outbreaks [23,24].

Improving Sanitation and Hygiene Practices

Improving sanitation and hygiene practices is another important strategy for preventing hepatitis A outbreaks in children. This includes promoting hand washing with soap and water, ensuring access to clean water and sanitation facilities, and encouraging safe disposal of human waste. Improving sanitation and hygiene practices can reduce the spread of hepatitis A by limiting the transmission of the virus through contaminated food and water and reducing the risk of person-to-person transmission [25,26].

Food Safety and Inspection

Ensuring the safety of food and water sources is also critical for preventing hepatitis A outbreaks in children. This includes regularly inspecting food establishments and water treatment facilities and enforcing strict food safety regulations. Proper food handling and preparation practices can also reduce the risk of contamination with the hepatitis A virus [27].

Health Education and Community Outreach Programs

Health education and community outreach programs can also play a key role in preventing hepatitis A outbreaks in children. These programs can educate parents, caregivers, and children about the risks and symptoms of hepatitis A and promote the importance of vaccination, proper hand hygiene, and safe food and water practices. Outreach programs can also target high-risk populations, such as those living in areas with poor sanitation and hygiene practices or areas affected by natural disasters [28].

The implications for public health are clear: hepatitis A outbreaks in children can be prevented through a comprehensive approach that includes vaccination, improving sanitation and hygiene practices, food safety and inspection, and health education and community outreach programs. Public health officials must collaborate with healthcare providers, food establishments, and water treatment facilities to implement these measures and reduce the incidence of hepatitis A outbreaks in children [29].

Despite significant progress in preventing hepatitis A outbreaks in children, further research is needed to understand the epidemiology of the disease better, identify high-risk populations, and develop new prevention strategies. Future research should focus on developing new vaccines, investigating new potential sources of infection, and evaluating the effectiveness of existing preventive measures. By continuing to invest in research, we can improve our understanding of hepatitis A outbreaks in children and develop more effective strategies to prevent them.

## Conclusions

In conclusion, hepatitis A outbreaks in children are a serious public health concern that can have long-term health consequences. Preventing these outbreaks requires a comprehensive approach that includes vaccination, improving sanitation and hygiene practices, food safety and inspection, and health education and community outreach programs. By implementing these measures, we can reduce the incidence of hepatitis A outbreaks in children and protect their health and well-being. Future research is needed to understand the epidemiology of the disease better and develop more effective prevention strategies.
